# Hyperspectral Imaging of the Hemodynamic and Metabolic States of the Exposed Cortex: Investigating a Commercial Snapshot Solution

**DOI:** 10.1007/978-3-319-91287-5_3

**Published:** 2018-04-16

**Authors:** Luca Giannoni, Frédéric Lange, Andrew L. Davies, Alisha Dua, Britta Gustavson, Kenneth J. Smith, Ilias Tachtsidis

**Affiliations:** 9 Department of Medical Physics and Biomedical Engineering, University College London, London, UK; 10 Department of Neuroinflammation, Institute of Neurology, University College London, London, UK

## Abstract

Hyperspectral imaging (HSI) systems have the potential to retrieve in vivo hemodynamic and metabolic signals from the exposed cerebral cortex. The use of multiple narrow wavelength bands in the near infrared (NIR) range theoretically allows not only to image brain tissue oxygenation and hemodynamics via mapping of hemoglobin concentration changes, but also to directly quantify cerebral metabolism via measurement of the redox states of mitochondrial cytochrome-c-oxidase (CCO). The aim of this study is to assess the possibility of performing hyperspectral imaging of in vivo cerebral oxyhemoglobin (HbO_2_), deoxyhemoglobin (HHb) and oxidized CCO (oxCCO) using commercially available HSI devices. For this reason, a hyperspectral snapshot solution based on Cubert GmbH technology (S185 FireflEYE camera) has been tested on the exposed cortex of mice during normoxic, hypoxic and hyperoxic conditions. The system allows simultaneous acquisition of 138 wavelength bands between 450 and 998 nm, with spectral sampling and resolution of ~4 to 8 nm. From the hyperspectral data, relative changes in concentration of hemoglobin and oxCCO are estimated and hemodynamic and metabolic maps of the imaged cortex are calculated for two different NIR spectral ranges. Spectroscopic analysis at particular regions of interest is also performed, showing typical oxygen-dependent hemodynamic responses. The results highlight some of the potentials of the technology, but also the limitations of the tested commercial solution for such specific application, in particular regarding spatial resolution.

## Introduction

Hyperspectral imaging (HSI) is an optical modality that involves the acquisition of images at numerous contiguous wavelength bands across a broad portion of the Electromagnetic spectrum Hyperspectral imaging (HSI). Such images then form a three-dimensional (3D) spatio-spectral dataset (*x*, *y*, *λ*), known as *hypercube* Hypercubes, where a complete spectrum is associated with each pixel of the imaged target [1].

In recent years, HSI has emerged as a promising imaging technology for biomedicalBiomedical optics applications and life science research, targeting different biological processes and several types of tissues [1]. In particular, it has shown the capability to retrieve quantitative information on Hyperspectral imaging (HSI) in both small animals and humans, by measuring changes in reflected light intensity at different spectral bands, in the visible and near-infrared (NIR) range [2, 3]. These intensity changes can be tracked back to modifications in the absorption properties of the imaged tissues during changes in oxygenation and blood perfusion, due to variations in the relative concentrations of the two states of Hemoglobin Hyperspectral imaging (HSI), i.e. oxygenated (HbO_2_) and deoxygenated (HHb). Furthermore, HSI could also potentially be used to monitor in vivo cerebral metabolism, by targeting a third chromophore that takes part in cellular respiration and energy production, namely Cytochrome-c-oxidase (CCO) and its redox forms [4]. This is possible via the detection of the optical spectral signature of the copper Cu_A_ redox centre of CCO, which is predominant in the NIR range between 780 and 900 nm [4, 5]. It has been also demonstrated [6, 7] that employing a large number of wavelengths over such relatively broad spectral range significantly improves the Signal-to-noise ratio (SNR) of the data and enhances discrimination between the CCOCytochrome-c-oxidase (CCO) and hemoglobin signals. Thus, HSI appears as particularly suited for imaging the hemodynamic and metabolic states of the brain, such as the exposed cerebral cortex of small animals.

In this study, the feasibility and potential of HSI for brain hemodynamicBrain hemodynamics and metabolism and metabolic monitoring are explored. In particular, the performance of a commercial snapshot hyperspectral solution is evaluated, focusing on its suitability to be employed on the exposed cerebral cortex of mice.

## Materials and Methods

A hyperspectral snapshot cameraHyperspectral imaging (HSI) based on Cubert GmbH technology (the S185 FireflEYE [8]) was chosen for the study, due to its technical characteristics: primarily, the number of wavelength bands (138) and its spectral resolution (~4 to 8 nm) and sampling (4 nm). The specifications of the camera are summarized in Table 1.

**Table 1:** Technical characteristics of the S185 FireflEYE snapshot camera [8]

Specifications	Cubert S185 FireflEYE
Acquisition mode:	Snapshot
Spatial resolution:	1000 × 1000 pixels
Spectral range:	450–998 nm
Spectral bands:	138
Spectral sampling:	4 nm
Spectral resolution (FWHM):	8 nm at 532 nm
Hypercube rate	Up to 5 hypercubes/s

The snapshot acquisition mode for HSI involves the simultaneous collection of every image at each spectral band, within a single integration time of the detector [1, 9]. This approach is preferred due to the benefits it provides for in vivo imaging, i.e. fast acquisition rate and low susceptibility to motion artifacts [9]. The S185 FireflEYE camera performs the snapshot acquisition mode by employing two charge-coupled device (CCD)Hyperspectral imaging (HSI) detectors, together with a pinhole and prisms for spatial and spectral separation, respectively. The spatial and the spectral information are then combined by an interpolation algorithm to obtain the final hypercubeHypercubes.

**Fig. 1:**
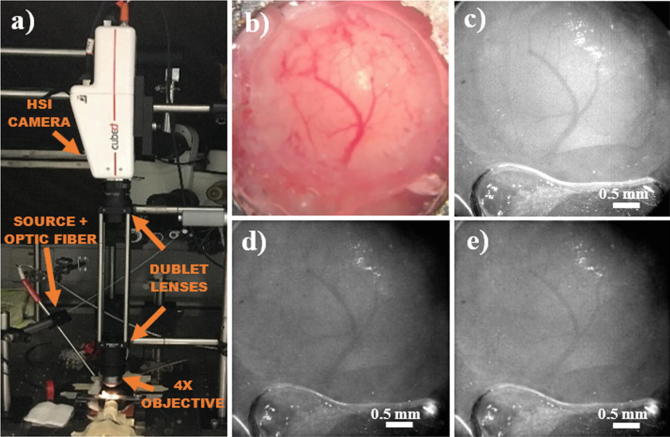
(**a**) Picture of the setup used for the investigation with the hyperspectral snapshot solution, highlighting the major components; (**b**) RGB microscope picture of the exposed cerebral cortex; (**c**) Spectrally averaged image of the same cortex, acquired during normoxic baseline; (**d**) hyperspectral image of the exposed cortex at 650 nm; (**e**) hyperspectral image of the exposed cortex at 750 nm

The setup used for the HSI study on the exposed cortex of mice is shown in Fig. 1a. A 4× achromatic objective coupled with two achromatic doublet lenses are implemented in front of the S185 FireflEYE snapshot camera, in order to achieve a Field of view (FOV) of about 4 × 4 mm. A broadband white light source (Ocean Optics HL-2000-FHSA) was then utilised for the illumination, directing it with a fibre optic needle. The FOV and spatial resolution of the setup were then estimated by imaging a calibrated spatial resolution phantom.

The hyperspectral setup described above was tested on the exposed cerebral cortex of anesthetized (1.5% isoflurane) healthy mice breathing normoxic, hyperoxic and several hypoxic gas mixtures in oxygen and nitrogen. These oxygen-dependent levels were established by manipulating the fraction of inspired oxygen (FiO_2_)Fraction of inspired oxygen (FiO2) of the mice. The experimental protocol consisted of the following consecutive phases: normoxic baseline (FiO_2_ = 21%); hyperoxia (FiO_2_ = 100%); normoxia (FiO_2_ = 21%); hypoxia (FiO_2_ = 15%); normoxia (FiO_2_ = 21%); hypoxia (FiO_2_ = 10%); normoxia (FiO_2_ = 21%); hypoxia (FiO_2_ = 5%); and death (FiO_2_ = 0%). During the normoxic baseline, 400 Hypercubes were acquired for 2 min, at 0.3 s delay between each cube. For each of the following phases, 1000 Hypercubes were acquired for 5 min, again at 0.3 s delay. Complete hypercubes for both the white reference, using a white reflectance standard, and the dark reference, by covering the lens of the camera, were also recorded using the same exposure times used for the experimental phases.

After data collection, the acquired Hypercubes of each phase and of the white reference were corrected by subtracting the dark reference hypercube from each of them. Normalization to the white reflectance was then performed for each of the hypercubes of the exposed cortex, by dividing them for the white reference hypercube. The corrected hypercubes were then integrated over time, to improve SNR. The 400 hypercubes of the normoxic baseline were time-averaged over their whole 2-min acquisition time. For the 1000 Hypercubes of each following phase, time-averaging was performed for every 100 hypercubes, obtaining ten time-averaged hypercubes per each condition (every 30 s during the experimental phases).

Finally, the time-averaged hypercubesHyperspectral imaging (HSI) were used to estimate the 2D maps of the relative changes in concentrations (per unit pathlength) Δ[HbO_2_], Δ[HHb] and Δ[oxCCO] at different time intervals. These maps were calculated by applying, pixel by pixel, the modified Beer-Lambert’s law [10], assuming a unitary differential pathlength (*DPF* = 1). Reference spectra for the molar extinction coefficients of hemoglobin and the oxidized-reduced molar extinction spectrum for CCOCytochrome-c-oxidase (CCO) were all obtained from UCL Biomedical Optics Research Laboratory (BORL) database [11].

Two sets of maps were obtained using the hyperspectral data at different spectral ranges: (1) between 778 and 902 nm (32 wavelength bands), aiming at resolving both the hemodynamic and the metabolic response from the HbO_2_, HHb and CCOCytochrome-c-oxidase (CCO) signals; and (2) between 650 and 986 nm (85 wavelength bands), considering only the hemoglobin signal, as to enhance visualisation of the hemodynamic response. The full range of the camera was not utilised for this last calculation due to the excessive amount of noise in the hypercubes below 650 nm. Differential spectroscopy analysis was also conducted on Regions of interest (ROI) of the exposed cortex, in particular within the major vessels and the surrounding tissue. This was achieved by spatially averaging the concentration changes across all the selected ROIs in the two sets of maps, in order to obtain the averaged temporal variations of Δ[HbO_2_], Δ[HHb] and Δ[oxCCO] in these ROIs during the different phases.

## Results

Figure 1b shows a Red-blue-green (RGB) picture of the exposed cortex of one of the mice, taken with a surgical microscope. Figure 1c presents the monochrome image obtained from the HSI setup during normoxic baseline, by spectrally integrating all the wavelength bands (138) in the hypercubeHypercubes. The exposure time for this image was set to 40 ms. It can be seen that the monochrome image provides good spatial resolution and details, compared with the colour image of the cortex: both major vessels (estimated size of ~150 μm), as well as the smaller ones (~50 to 60 μm), are resolved. However, by looking at the images at single spectral bands (as shown in Fig. 1d, e, depicting the target at 650 and 750 nm, respectively), image contrast appears considerably lower, with only the major vasculature resolved. The images at single bands also display a significant amount of noise. Furthermore, the reflectance spectra of the pixels of the Hypercubes present less smooth profiles compared with the typical intensity spectra obtained in broadband NIR spectroscopy.

**Fig. 2:**
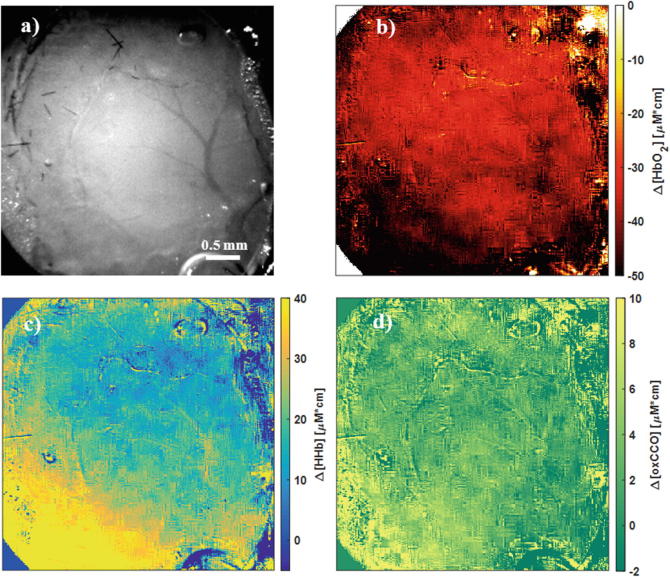
Maps of the relative concentration changes (per unit pathlength) in HbO_2_ (**b**), HHb (**c**) and oxCCO (**d**) in the exposed cerebral cortex during transition from hypoxia to death (FiO_2_ from 5% to 0%), analysed in the spectral range between 778 and 902 nm. The maps are compared with the spectrally averaged image of the same cortex, acquired during normoxic baseline (**a**)

To try to overcome these issues, the exposure time was increased to 70 ms for the following mouse, pushing the intensity of the reflected light almost to the saturation value of the camera, in order to increase image SNR as much as possible. Results of Δ[HbO_2_], Δ[HHb] and Δ[oxCCO]Cytochrome-c-oxidase (CCO) for such configuration in the range 778–902 nm are shown in Fig. 2, for the last hypoxic condition (FiO_2_ from 5% to death). Image contrast in these maps is low: however, the time progression of the hemodynamic response on the major vessels can be localised for HbO_2_ and HHb, although it appears blurred and spread also outside the vasculature. The map of oxCCO results even much noisier and only a very slight localisation of the metabolic response is possible. Spatial resolution is also limited to indistinct shapes of the vasculature.

**Fig. 3:**
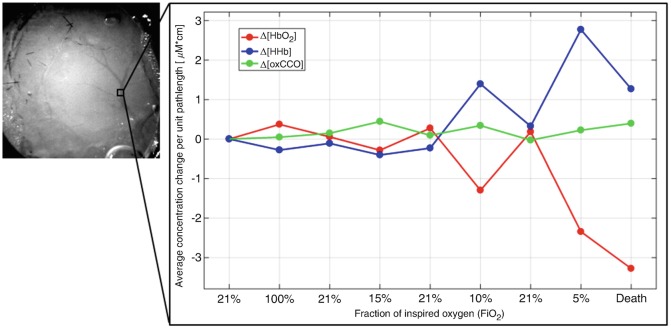
Spectroscopic analysis of a ROIRegions of interest (ROI) including a major vessel of the exposed cortex, showing the temporal changes Δ[HbO_2_] (red), Δ[HHb] (blue) and Δ[oxCCO] (green), in the spectral range between 778 and 902 nm, during all the different oxygen-dependent conditions of the experiment

**Fig. 4:**
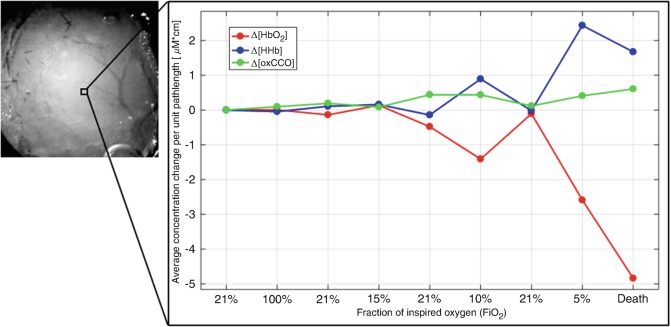
Spectroscopic analysis of a ROIRegions of interest (ROI) in the tissue outside major vessels of the exposed cortex, showing the temporal changes Δ[HbO_2_] (red), Δ[HHb] (blue) and Δ[oxCCO] (green), in the spectral range between 778 and 902 nm, during all the phases of the experiment

The spectroscopic analysis of the ROIRegions of interest (ROI) inside the major vasculature (Fig. 3) provides results consistent with the expected hemodynamic response of HbO_2_ and HHb for all the phases. The magnitude of this response is considerably lower for the ROI in the surrounding tissue (Fig. 4), as predicted, and it only increases in the last phases, possibly because of spreading outside the vessels. Changes in oxCCO are observed in both ROIs (even the one within the vessels), although due to the significant amount of noise in the corresponding map, it is difficult to attribute these changes to actual physiological and metabolic variations in the brain tissue.

**Fig. 5:**
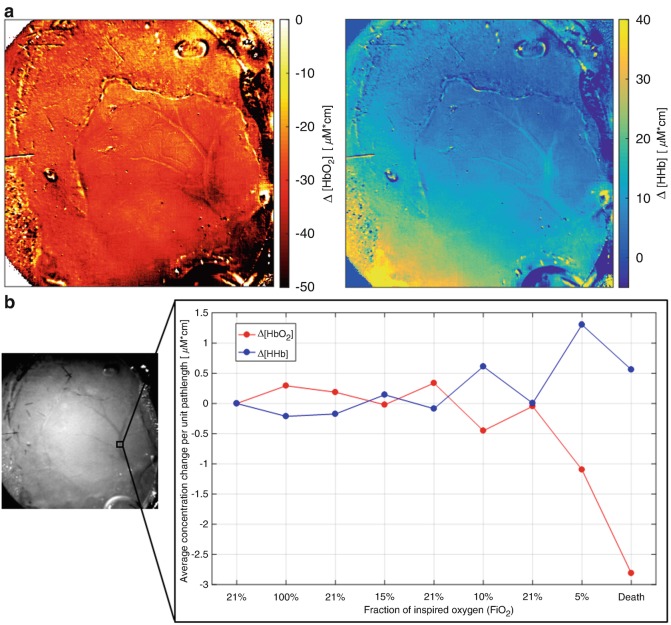
(**a**) Recalculated hemodynamic maps of Δ[HbO_2_] (left) and Δ[HHb] (right) for the spectral range 650–986 nm. (**b**) Spectroscopic analysis of a ROI from the above maps that includes a major vessel of the exposed cortex, showing the temporal changes of Δ[HbO_2_] (red) and Δ[HHb] (blue)

Finally, Fig. 5a presents the calculated maps of only Δ[HbO_2_] and Δ[HHb] in the broader NIR range (650–986 nm). Spatial resolution of these maps appears significantly higher, with detailed visualisation of the larger vasculature, as well as of the smaller one. The hemodynamic response is also more clearly resolved and localised, especially for HHb. This is supported by the expected results of the related spectroscopic analysis of the ROI inside the major vessels, as reported in Fig. 5b.

No quantification of the depth of the measurements in the brain tissue was possible, due to lack of estimate of the differential optical pathlength, as mentioned.

## Discussion and Conclusions

The results of the study have highlighted some of the major advantages of HSI technologies and of the commercial snapshot solution, but some of the drawbacks and limitations of the tested instrument for in vivo brain metabolic monitoring in small animals are also apparent. In particular, the tested HSI solution has shown its capability to localise the hemodynamic response in the exposed cortex from all the maps. Furthermore, the Hyperspectral imaging (HSI) retrieved correct time-varying hemodynamic information from the exposed cortex under different oxygen-dependent conditions. However, although the spatial resolution of the maps was adequate for the range 650–986 nm, it was not high enough for imaging all the vasculature details in the selected range for the CCOCytochrome-c-oxidase (CCO) signal (778–902 nm). Better focusing and magnification may be necessary to achieve this goal and to improve image quality. Therefore, this limited spatial resolution makes impossible to assess the correct evaluation of the metabolic response. Indeed, a better spatial resolution is required to minimize image noise, in order to discern the focalised and smaller changes in oxCCO (about ten times lower than hemoglobin) in the specific NIR wavelength interval.

Furthermore, the selection of the spectral range for the metabolic analysis of CCO is fundamental, since different redox complexes of CCOCytochrome-c-oxidase (CCO) contribute differently to its optical signatures in the visible and NIR range: among them are the heam iron centres (cytochrome a and a_3_), the copper Cu_B_ centre, as well as intermediate oxygenated forms of CCO (namely P and F) [5]. The aforementioned compounds also show different responses to metabolic changes. Accurate HSI analysis of these spectral signatures could provide a more comprehensive overview of brain metabolism.

Considering the previous points, the current commercial HSI setup was found not suitable for in vivo metabolic monitoring, without some customising steps. Therefore, we are currently investigating bespoke solutions for HSI of the exposed cortex.
